# An NO/GSNO-based Neuroregeneration Strategy for Stroke Therapy

**Published:** 2015-12-31

**Authors:** Mushfiquddin Khan, Tajinder Singh Dhammu, Tejbir Singh Dhaindsa, Hamza Khan, Avtar K Singh, Inderjit Singh

**Affiliations:** 1 Department of Pediatrics, Medical University of South Carolina, Charleston, SC, USA; 2 Faculty of Medicine, University of South Carolina, Columbia, SC, USA; 3 Department of Pathology and Laboratory Medicine, Medical University of South Carolina, Charleston, SC, USA

**Keywords:** Stroke, GSNO, Neuroregeneration, S-Nitrosylation, Functional recovery, HIF-1α, Angiogenesis

## Abstract

Stroke is associated with significant morbidity and mortality due to the limited neuroregeneration capacity of the injured brain. Other than thrombolysis in the acute phase of the disease by tissue plasminogen activator (tPA), which offers only a short window of treatment (~3 hours), an ideal stroke therapy is not available mainly because of limited understanding of the mechanisms of neuroregeneration and functional recovery in the chronic phase. Yet many drug therapies, including S-nitrosoglutathione (GSNO), have been shown to provide neuroprotection against acute disease in animal models of transient cerebral ischemia reperfusion (IR) and permanent ischemia. GSNO was also effective in stimulating neuroregeneration-related factors in the chronic phase of the disease. In this short review, we assess the evidence supporting exogenous administration of GSNO after experimental stroke as a means to stimulate neuroregeneration and aid in functional recovery via stabilization of the HIF-1α/VEGF pathway.

## Introduction

Stroke is the leading cause of serious, long-term disability in the USA at great burden to families and medical care agencies. Furthermore, it is associated with significant morbidity/mortality and a high cost of approximately $50–65 billion annually in the USA [1]. In developed countries, the incidence of stroke is declining, largely due to successful efforts at lowering blood pressure and reducing smoking. However, the overall rate of stroke remains high due to increases in aging populations. Nearly three-quarters of all strokes occur in people over the age of 65. The risk of having a stroke more than doubles each decade after the age of 55 years. Therefore, age is recognized as the most significant non-modifiable risk factor for stroke. In the aged population, the mechanisms of stroke disease are distinct from stroke mechanisms in the younger population. Older stroke survivors have not only compromised neuroprotection mechanisms but also slower rates of neuroregeneration. Improvement by a therapeutic agent of these rates of neuroregeneration and neurobehavioral functions will determine the efficacy and the clinical relevance of a therapy in stroke. Neuroregeneration mechanisms are significantly dependent on the reduction of nitro-oxidative stress, and these mechanisms are reported to be stimulated by GSNO following stroke injury. Therefore, the aim of this article is to highlight that GSNO stimulates neuroregeneration mechanisms via the stabilization of the HIF-1α/VEGF pathway in the chronic phase of stroke disease.

GSNO is a natural component of the human body produced by the reaction of NO with glutathione (GSH) in the presence of oxygen [2]. It is present in the brain and other organs [3]. GSNO is directly involved in cell signaling via S-nitrosylation of target proteins, including NF-κB, STAT3, COX-2, caspase-3, calpains, inducible nitric oxide synthase (iNOS), and endothelial NOS [4–7]. Exogenous administration of GSNO [8] also protects against cardiac ischemic injury [9,10], supporting the therapeutic potential of GSNO. Pharmacological inhibition of GSNO reductase (GSNOR) has also been shown to improve endothelial functions [11], indicating a protective role of GSNO in neurovascular dysfunction-related diseases. Studies have reported that GSNO inhibits platelet activation in humans [12] and protects both BBB integrity and epithelial permeability [13,14].

In a microenvironment of stroke injury, NO released by conventional NO-donors or NO gas itself is immediately inactivated by superoxide, forming peroxynitrite. This disadvantage of inactivation is not associated with the S-nitrosylating agent GSNO. In addition, S-nitrosylation of cysteine residue (a reversible modification) prevents it from further oxidation to sulfinic and sulfonic acids (an irreversible modification), thereby preventing inactivation of both NO and proteins. Neurorepair from GSNO may be mediated by two different mechanisms: 1) S-nitrosylation and 2) maintaining redox by mechanistically reducing the production of oxidants, including peroxynitrite. This multi-mechanistic functional and therapeutic potential is not embedded in conventional NO donors, making GSNO a unique candidate to be investigated for the stimulation of functional recovery following stroke. Furthermore, GSNO therapy can be initiated even late with or without the administration of the FDA-approved tissue plasminogen activator (tPA). GSNO is inexpensive, readily available, and GSNO’s exogenous administration in humans or animals has not resulted in toxicity or side effects.

GSNO activates Akt via S-nitrosylation-dependent inhibition of PTEN [15]. PTEN is a lipid phosphatase, which regulates cellular phosphatidylinositol phosphate levels and PI3 kinase signaling. S-nitrosylation of PTEN results in inhibition of its activity, leading to the activation of Akt [16]. Inhibition of GSNO-mediated Akt activation reverses GSNO-mediated neuroprotection and functional recovery [17]. Activation of Akt is reported to stabilize hypoxia-inducible factor-1 alpha (HIF-1α), which, in turn, induces VEGF and angiogenesis [18]. HIF-1α is also stabilized by its direct S-nitrosylation, and S-nitrosylation-mediated stabilization of HIF-1α has been reported to increase angiogenesis in a myocardial injury model [10], indicating an overall regenerative role of S-nitrosylated HIF-1α. There is a close relationship between angiogenesis/neurogenesis and VEGF. Increased neurogenesis is accompanied by increased angiogenesis, whereas angiogenesis up regulates neurotrophic factors. Angiogenesis itself is regulated by VEGF, mainly via HIF-1-based transcription.

**Figure 1:**
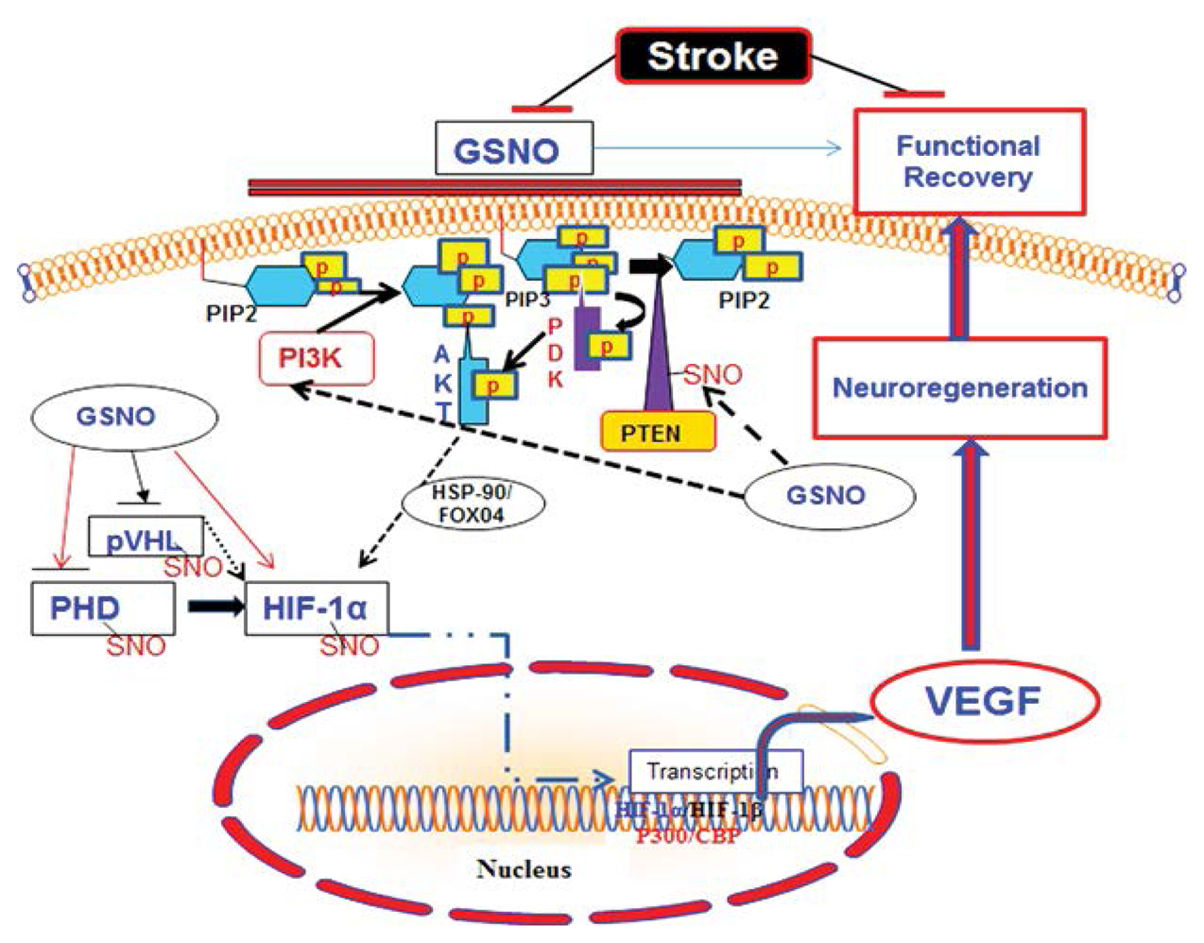
Hypothesized GSNO-mediated events leading to stimulation of neuroregeneration and functional recovery. GSNO levels are decreased, and functional recovery is compromised following stroke. S-nitrosylation by GSNO of PTEN causes its inhibition, resulting in activation of Akt. Activated Akt inhibits PHDs such as PHD3, thus blocking the binding of HIF-1α with pVHL and stabilizing HIF-1α, which is S-nitrosylated by GSNO, leading to its further stabilization, translocation to nucleus, and dimerization with HIF-1β. These mechanisms facilitate recruitment of P300/CBP and transcribe VEGF. VEGF, in turn, stimulates regeneration (angiogenesis), leading to neurogenesis and functional recovery following stroke.

Hippocampal neuronal cell loss following IR is linked to cognitive alterations, leading to impairments in learning and memory. The adult mammalian brain generates new neurons (neural progenitor cells; NPC) in the hippocampus, dentate gyrus (DG), and subventricular zone (SVZ). These progenitors possess the ability to self-renew, differentiate into mature neurons, and enhance plasticity. Restorative neurogenesis occurs after stroke, but a majority of the new cells die as a result of the oxidative environment, resulting in limited and insufficient functional recovery. Therefore, the focus of functional recovery research in stroke is dual pronged: the induction of neurogenesis and protection against oxidative/inflammatory loss [19]. GSNO treatment of stroke confers not only this neuroprotection against neuroinflammatory insults but also induces angiogenesis via stimulating VEGF through the stabilization of the HIF-1α/VEGF pathway. VEGF has been shown to modulate coupling of angiogenesis and neurogenesis; hence, it is essential for neuroregeneration [20]. GSNO-mediated increased cell proliferation and vessel density correlating with functional recovery in a 2-week IR study [21] further support the neurodegenerative role of GSNO. Based on neuroprotective efficacy and neuroregenerative ability, GSNO has strong potential as a drug candidate for evaluation in human stroke. However, certain precautions are associated with the use of GSNO as therapy due to its structural configuration. GSNO is comparatively less stable compound and its metabolism is affected by several factors including light, temperature, metals and enzymes [22]. The activity of GSNO is also associated with hypotension [23]; therefore, physiologic parameters must be monitored if higher doses of GSNO are used. In our studies, freshly prepared (in dark) GSNO (1–3 μM/kg body weight) is slowly (10–15 min) infused in experimental animals via jugular vein or tail vein. This protocol of GSNO preparation and administration had no any significant change in physiologic parameters [24]. Currently, we are investigating the efficacy of GSNO for functional recovery in permanent ischemic stroke animal models (Figure 1).

## Conclusion

HIF-1α stabilization-based intervention in ischemia using inhibitors of its hydroxylating enzyme prolyl hydroxylases (PHDs) has provided neuroprotection and stimulated neurorecovery in a number of preclinical studies [25], indicating that HIF-1α stabilization by GSNO/S-nitrosylation is a logical target for stroke treatment. GSNO is an endogenous neuroregeneration-inducing agent, and its exogenous administration protects against both acute and chronic phase injuries following experimental stroke. Furthermore, no toxicity or side effects were reported following its administration in humans [12,26,27] for other indications or in animals [28]. Therefore, investigating the potential of S-nitrosylation mechanism using GSNO as a therapeutic agent in stroke is a promising approach with clinical implications. We propose that an administration of low dose GSNO is an ideal strategy to stimulate neuroregeneration mechanisms for functional recovery in stroke patients.
